# Editorial: Plant defense mechanisms in plant-pathogen interactions

**DOI:** 10.3389/fpls.2023.1292294

**Published:** 2023-11-21

**Authors:** Huan Peng, Hongjie Feng, Tao Zhang, Qi Wang

**Affiliations:** ^1^ State Key Laboratory for Biology of Plant Diseases and Insect Pests, Institute of Plant Protection, Chinese Academy of Agricultural Sciences, Beijing, China; ^2^ National Key Laboratory of Cotton Biology, Institute of Cotton Research, Chinese Academy of Agricultural Sciences, Anyang, China; ^3^ Institute of Microbiology, Chinese Academy of Sciences, Beijing, China; ^4^ Department of Plant-Microbe Interactions, Max Planck Institute for Plant Breeding Research, Cologne, Germany

**Keywords:** plant signaling, hormone signaling, plant-pathogen interactions, plant immune systems, resistance gene, plant secondary metabolite

Plant pathogens cause serious damage to crop production and pose a threat to agriculture and natural ecosystems. A profound understanding of plant-pathogen interactions is vital for developing innovative disease control and environmental protection strategies in crop production (Bulasag et al.). Despite decades of dedicated research unraveling plant immunity intricacies, comprehending the complex cross-kingdom interactions among diverse hosts and microorganisms remains challenging. This Frontiers e-book on “Plant Defense Mechanisms in Plant pathogen Interactions” provides 19 articles covering research on various mechanisms between plants and pathogens. The aim of this summary is to offer a fresh perspective and new insight into the intricate mechanisms governing plant immunity within a spectrum of plant-pathogen interactions.

Ge et al. conducted a study on the role of the *Brrpp1* gene in *Plasmodiophora brassicae*-infected Chinese cabbage, aiming to understand its significance in combating clubroot disease. This study revealed that *Brrpp1* contains the typical TIR-NBS-LRR domain of an R gene. While the gene expression of *Brrpp1* remained unaffected by *P. brassicae* infection, differences in the cDNA and promoter sequences resulted in alterations in protein structure and significant variations in gene expression between resistant and susceptible materials. In another study on the interaction between *Cladosporium fulvum* and tomato, Peng et al. discovered that several core defense genes and the plant hormone pathways were affected by *C. fulvum* inoculation.

The antagonistic effects of salicylic acid (SA) and jasmonate acid (JA)-mediated defense signaling pathways during pathogen invasion are well-known (Thaler et al., 2012). The study conducted by Li et al. revealed that the inhibition of WRKY53 resulted in the suppression of the SA pathway and activation of the methyl jasmonate (MeJA) pathway, leading to reduced plant resistance against *Verticillium dahliae*. This modulation of MeJA pathway-related gene expression has the potential to alter the tolerance of upland cotton towards *V. dahliae*. The research conducted by Yang et al. revealed that lipopeptide mycosubtilin homologues from *Bacillus subtilis* BS-Z15 are capable of functioning as inducers to activate the expression of ethylene (ET) and JA-related defense genes, thereby triggering systemic acquired resistance (ISR) in plants. Rice Sheath Flight (ShB) disease poses a significant threat to global rice production. Therefore, understanding the mechanisms that regulate resistance to ShB in rice is crucial for its management. Chen et al. summarizes the regulatory effects of hormones on ShB and the interplay between different hormones.

Sugar is an important nutrient that plants and pathogens both vie for. The competition between plants and microbes occurs in various ways, and Chen et al. briefly reviewed the mechanisms of sugar transport regulation. The competition’s result is influenced by the characteristics and specificities of sucrose and monosaccharide transporters in both the host and pathogen. By studying how proteins responsible for sugar transport function in pathogens with different sugar preferences, we can uncover immune-related regulatory pathways shared by pathogens with similar infection strategies, as well as a wider range of disease resistance strategies. Surprisingly, *V. dahliae* was found to have a series of sugar transporter genes, including *Vdst3* and *Vdst12*, which are essential for the growth and pathogenicity of *V. dahlia* (Chen et al.).

In the study by Qi et al., the researchers investigated the interaction between wheat leaf rust and wheat. They discovered that a protein called Pt13024, produced by the leaf rust fungus, effectively suppresses a type of cell death triggered by certain substances. This protein triggers a strong resistance response in the wheat, including the deposition of callose and the release of reactive oxygen species (ROS), resulting in a hypersensitive response. When Pt13024 is silenced, the virulence of another strain of the fungus is enhanced in the wheat. This suggests that Pt13024 is avirulent to the wheat variety TcLr30. In another study by Liu et al., a genome-wide analysis of wheat genes belonging to the LRR-RLK family was conducted after wheat mosaic virus infection. The researchers found that these genes play a role in various processes, indicating that they are important in conferring resistance to virus infestation in wheat through hormonal signaling. Additionally, Mapuranga et al. reviewed the interactions between wheat and a type of fungus called *Blumeria graminis* f. sp. *tritici* (Bgt), as well as the defense mechanisms of wheat against Bgt infection. The aim of this review was to provide a comprehensive understanding of controlling Bgt and highlight potential means of improving wheat resistance to this fungus.

The primary function of the root cell wall is to act as an initial physical and protective barrier against pathogens. Quiroz-Figueroa et al. investigated the response of different maize varieties to *Fusarium verticillioides* (Sacc.) Nirenberg (Fv) infection, revealing a novel maize root mechanism involved in pathogen infection. Similarly, Cai et al. performed RNA-seq on three plum varieties to uncover the defense mechanisms against Armillaria Root Rot (ARR) in Prunus rootstocks. Their findings highlighted several key components contributing to ARR resistance.

In their study, Bulasag et al. found that *Botrytis cinerea* can distinguish between phytoalexins and trigger specific gene expression, the efflux transporter BcatrB plays a crucial role in enabling *B. cinerea* to evade innate immune responses in various important crops. Zeng et al. have thoroughly reviewed current research on poplars, which not only serve as a model system for woody plants but also act as hosts for various fungi. Their study specifically investigates poplar’s defensive responses to both necrotrophic and biotrophic fungi. Furthermore, the review provides suggestions for enhancing poplar disease resistance and offers valuable perspectives for future research endeavors.

The research conducted by Wang et al. focuses on exploring the role of ECT9 in regulating plant immunity in *Arabidopsis*. The study reveals that ECT9 forms condensates with its homolog ECT1, and this condensation plays a crucial role in coordinating their functions. Among the 13 tested YTH family members, only ECT9 forms condensates that decrease in response to SA treatment. However, ECT1 can still be recruited to ECT9 condensates. Interestingly, the *ect1/9* double mutant exhibits enhanced immune responses to avirulent pathogens, suggesting that co-condensation serves as a mechanism for redundant functions among members of the RBP family. Phenylalanine ammonia lyase (PAL) is an essential enzyme in phenylpropane metabolism and plays a crucial role in response to biotic and abiotic stress. Zhang et al. investigated the expression of *Pal* genes in tomatoes when exposed to root-knot nematode infection. This study provides important insights into the notable expression patterns of *Pal* genes in tomatoes under the influence of biotic stresses, particularly root-knot nematodes.

D’Errico et al. discovered that the Tomato yellow leaf curl Sardinia virus (TYLCSV) has the ability to enhance tomato resistance to drought. The C4 protein of TYLCSV was found to play a crucial role in this resilience. Additionally, when the C4 protein is overexpressed, it also enhances the tomato’s resistance to powdery mildew. This improvement in resistance is likely due to the activation of a pattern-triggered immunity (PTI) response, which is influenced by the TYLCSV-C4 protein. The findings of this study significantly enhanced our understanding of how tomato expressing TYLCSV C4 can develop tolerance to powdery mildew.

Recently, scientists have made significant progress in unraveling the structures and functions of plant NLR proteins, including the Arabidopsis immune receptor ZAR1, a CC domain NLR (CNL) acting as a resistome to initiate immunity and cell death (Wang et al., 2019; Ma et al., 2020; Bi et al., 2021). Similarly, the wheat CNL protein Sr35 forms a resistome-like structure, the effector protein AvrSr35 directly binding to the C-terminal region of the LRR (Förderer et al., 2022). While TNL resistome stimulates the production of pRib-AMP and pRib-ADP, which binding to the EDS1-PAD4 complex to enhance immunity signaling (Huang et al., 2022; Jia et al., 2022). Additionally, TIR domain NLRs (TNLs) can convert RNA and/or DNA to cyclic nucleotide monophosphates (cNMPs) to induce cell death during defense (Yu et al., 2022). These findings imply that plants employ multiple immune mechanisms to adapt to complex environments. The lingering question is how the diverse plant immunity mechanisms could contribute to future advancements in crop production. Therefore, we believe that the utilization of cutting-edge technologies, such as structural biology and comparative analysis utilizing large-scale genome information, will significantly enhance our understanding of plant immunity in the future.

Similarly, all articles related to the Research Topic “*Plant defense mechanisms in plant-pathogen interactions*” provide insights into how plants combat pathogens and highlight the significance we place on controlling plant pathogens. The diverse strategies employed by plant pathogens to overcome plant defenses underscore the complex and dynamic nature of plant-pathogen interactions. Understanding these interactions and the underlying mechanisms is crucial for developing effective strategies to manage plant diseases in both agricultural and natural ecosystems in the future.

## Author contributions

HP: Writing – original draft, Writing – review & editing. HF: Writing – review & editing. TZ: Writing – review & editing. QW: Writing – review & editing.

## References

[B1] Bi G. Z. Su M. Li N. Liang Y. Dang S. Xu J. C. . (2021). The ZAR1 resistosome is a calcium-permeable channel. triggering plant immune signaling. Cell 184, 3528–3541. doi: 10.1016/j.cell.2021.05.003 33984278

[B2] Förderer A. Li E. Lawson A. W. Deng Y. N. Sun Y. Logemann E. . (2022). A wheat resistosome defines common principles of immune receptor channels. Nature 610 (7932), 532–539. doi: 10.1038/s41586-022-05231-w 36163289 PMC9581773

[B3] Huang S. J. Jia A. L. Song W. Hessler G. Meng Y. G. Sun Y. . (2022). Identification and receptor mechanism of TIR-catalyzed small molecules in plant immunity. Science 377 (6605), eabq3297. doi: 10.1126/science.abq3297 35857645

[B4] Jia A. L. Huang S. J. Song W. Wang J. L. Meng Y. G. Sun Y. . (2022). TIR-catalyzed ADP-ribosylation reactions produce signaling molecules for plant immunity. Science 377 (6605), eabq8180. doi: 10.1126/science.abq8180 35857644

[B5] Ma S. C. Lapin D. Liu L. Sun Y. Song W. Zhang X. X. . (2020). Direct pathogen-induced assembly of an NLR immune receptor complex to form a holoenzyme. Science 370 (6521), eabe3069. doi: 10.1126/science.abe3069 33273071

[B6] Thaler J. S. Humphrey P. T. Whiteman N. K. (2012). Evolution of jasmonate and salicylate signal crosstalk. Trends Plant Sci. 17 (5), 260–270. doi: 10.1016/j.tplants.2012.02.010 22498450

[B7] Wang J. C. Hu M. J. Wang J. Qi J. F. Han Z. F. Wang G. X. . (2019). Reconstitution and structure of a plant NLR resistosome conferring immunity. Science 364 (6435), eaav5870. doi: 10.1126/science.aav5870 30948527

[B8] Yu D. L. Song W. Tan E. Y. J. Liu L. Cao Y. Jirschitzka J. . (2022). TIR domains of plant immune receptors are 2’,3’-cAMP/cGMP synthetases mediating cell death. Cell 185 (13), 2370–2386.e18. doi: 10.1016/j.cell.2022.04.032 35597242

